# PIK3CA and p53 Mutations by Next Generation Sequencing in Lymphoepithelioma-Like Carcinoma of the Endometrium

**DOI:** 10.1155/2018/5894589

**Published:** 2018-05-03

**Authors:** Lucie Bienfait, Nicky D'Haene, Xavier Catteau, Jean-Christophe Noël

**Affiliations:** Department of Pathology, Erasme University Hospital/Curepath, Free University of Brussels (ULB), Brussels, Belgium

## Abstract

Lymphoepithelioma-like carcinoma of the endometrium is a very rare variant of endometrial carcinoma characterized by syncytial nests of pleomorphic epithelial cells and heavy infiltration of the stroma by lymphocytes (in particular CD8 cytotoxic T-lymphocytes) and plasma cells. Until now, only five cases have been characterized in this location. This report describes the clinicopathological and the molecular features of this unusual tumor. In particular, using the next generation sequencing (NGS) technique, we have demonstrated that this tumor could be associated with PIK3CA and p53 gene mutations. These data have not been reported to date and suggest that lymphoepithelioma-like carcinoma of the endometrium shares common molecular features with high grade endometrioid and serous-like endometrial carcinoma which are associated with poor outcome. Nevertheless, in endometrial lymphoepithelioma-like carcinoma, the alterations on cell cycle, apoptosis, and/or senescence secondary to p53 mutations could potentially be counterbalanced by the antitumoral response induced by CD8 cytotoxic T-lymphocytes numerous in these tumors.

## 1. Introduction

Lymphoepithelioma-like carcinoma is an unusual variant of carcinoma characterized by poorly defined nest of epithelial cells closely intermixed with abundant lymphoid infiltrate. This entity has been primary described in the head and neck [1–3]. In the genital tract, this pathological entity is very rare, mainly described in the cervix, where it could be associated with Human Papillomavirus (HPV) infection [4–7]. Exceptionally, these tumors have been also encountered in the rest of genital tract including vulva, vagina, ovary, and endometrium [8–11]. In the endometrium, to the best of our knowledge, only 5 cases of lymphoepithelioma-like carcinoma have been reported [12–16]. Recently, improvements in genomic profiling have highlighted endometrial carcinoma subclassification with at least four main groups including (1) POLE (a gene which encodes the catalytic subunit of DNA polymerase epsilon) ultramutated tumors, (2) MSI (microsatellite instable)/hypermutated tumors with MLH1 (MulL homolog 1) promoter methylation and subsequent frequent loss of the protein by immunohistochemistry, (3) MSS (microsatellite-stable) with low mutations of different genes and in particular CTNNB1 gene (catenin beta 1), and (4) copy number-high, serous-like tumors with frequent p53 mutations and strong nuclear p53 protein immunohistochemical expression [17–20]. However, until now, due to their rarity, no molecular genetic alterations have been described in lymphoepithelioma-like endometrial carcinoma. Therefore, the aim of the present study is to assess the molecular profile of this entity using the next generation sequencing (NGS) technique correlation with corresponding immunohistochemical data.

## 2. Case Presentation

This study was performed according to the rules of local ethics committee and to the Belgian legislation which provides that firstly no approval of ethical reference was necessary for a case presentation/case report and secondly that the patient consent for the use of residual human body material for scientific research purposes shall be deemed to have been given provided that the patient does not communicate their refusal (“opting out”) before any operation is carried out with this residual human body material. The anonymity of the patient without privacy and personally identifiable information was observed.

A 67-year-old woman was admitted in March 2016, to the Erasme University Hospital for irregular and abnormal vaginal bleeding occurring one year before. Her past medical history was characterized by severe obesity with a body mass index evaluated to 35, hypertension, and type 2 diabetes mellitus. Gynaecological examination was unremarkable but vaginal ultrasound and pelvic MRI control showed a corporal endocavitary mass of 25 × 17 mm relevant to endometrial neoplasia stage FIGO IA. Endometrial curettage was performed and revealed a poorly differentiated invasive carcinoma and therefore the patient underwent radical hysterectomy with bilateral salpingo-oophorectomy associated with a pelvic and lumbar-aortic lymphatic dissection.

**Figure 1:**
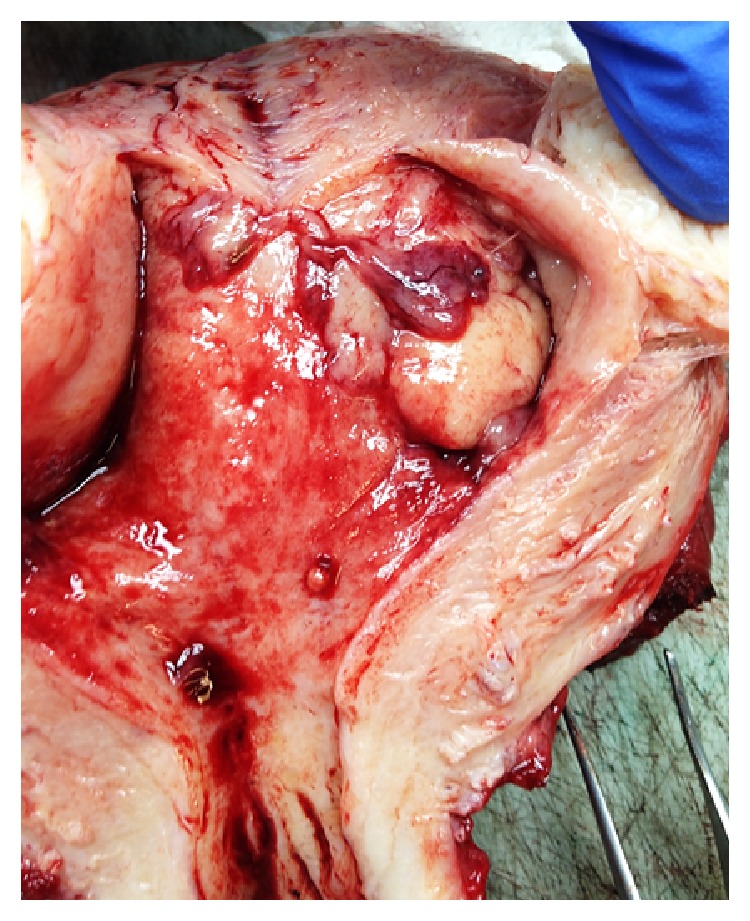
Macroscopic aspect of lymphoepithelioma-like carcinoma of the uterus. Polypoid tumor of 20 × 15 mm located in the fundus with areas of haemorrhage and necrosis.

Macroscopically, a partially polypoid mass of 20 × 15 mm, mainly located in the uterine fundus, was observed. The tumor was soft, with areas of haemorrhage and necrosis, and invaded less than half of the myometrium (Figure 1). The adnexa, parameters, and lymph nodes were macroscopically unremarkable.

**Figure 2:**
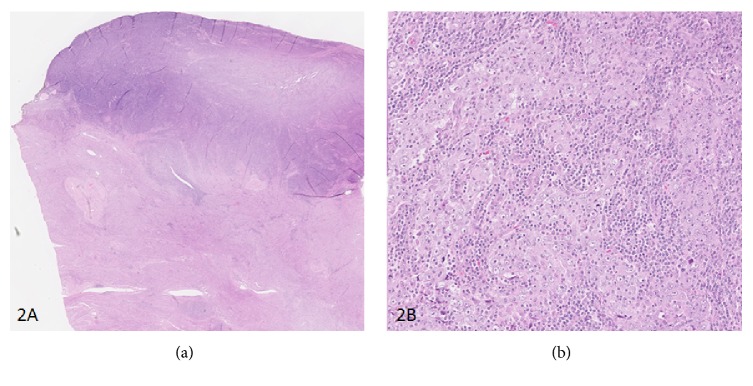
Microscopic aspects of lymphoepithelioma-like carcinoma. At low power view, the endometrial tumor appeared as “bluish” relatively well limited with focal myometrial invasion (a). At high power view, note the syncytial aspect of the tumor nests and heavy infiltration of the stroma by lymphocytes and plasma cells closely intermingled with epithelial cells (b).

Microscopically, the tumor consisted of syncytial tumor nests of pleomorphic epithelial cells often with large nuclei and prominent nucleoli. No or only minimal glandular differentiation was observed. The surrounding stroma was heavily infiltrated by lymphocytes and plasma cells with numerous lymphoepithelial complexes. An infiltration of the inner layers of the myometrium was observed (Figure 2).

**Figure 3:**
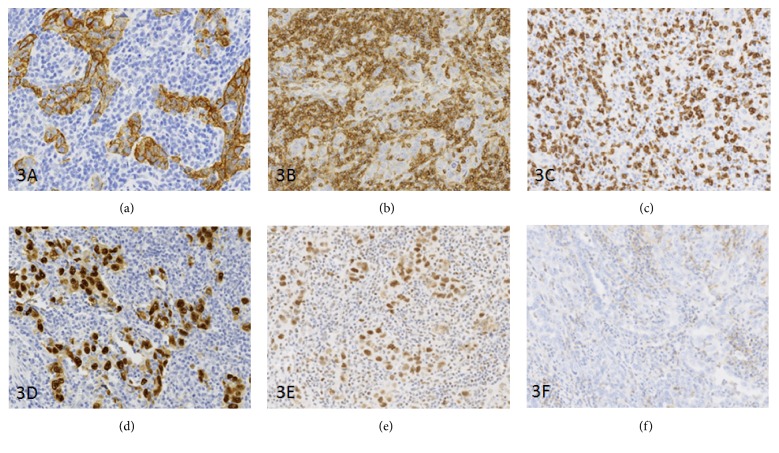
Immunohistochemical aspect of lymphoepithelioma-like carcinoma. Positivity of epithelial component for cytokeratin AE1/AE3 (a) and in mirror of the stromal lymphocytes for CLA (b). Note that CD8+ cytotoxic T-lymphocytes are numerous (c). Strong nuclear expression of tumor cells for the p53 (d) and lesser for MLH1 (e). PDL1 expression was restricted to some stromal lymphocytes but the epithelial cells were negative (f).

Using immunohistochemistry, as we have previously described, the carcinomatous component was positive for broad spectrum cytokeratins AE1/AE3 (clone* AE1/AE3, 1 : 150, Dako *Glostrup, Denmark), cytokeratin 7 (CK7) (clone* OV/TL12/30, 1 : 400, Leica* Newcastle, United Kingdom), estrogen (ER) (clone* EP1, 1 : 50, Dako *Glostrup, Denmark) and progesterone (PR) (clone* 16 + SAN27, 1 : 500, Leica *Newcastle, United Kingdom), Vimentin (vim) (clone* V9, 1 : 1000, Dako* Glostrup, Denmark), p53 protein (strong nuclear staining) (clone* DO-7, 1 : 200, Dako *Glostrup, Denmark), MLH1 (MutL homolog 1) (clone* ES05, 1 : 50, Leica *Newcastle, United Kingdom), MSH2 (MutS homolog 2) (clone* FE11, 1 : 50, Dako *Glostrup, Denmark), MSH6 (MutS homolog 6) (clone* EP49, 1 : 100, Dako *Glostrup, Denmark), and PMS2 (postmeiotic segregation 2) (clone* EP51, 1 : 100, Dako *Glostrup, Denmark) [21, 22] (Figure 3). Stromal lymphoid cell showed a CLA (clone* 2B11 + PD7/26, ready to use, Dako *Glostrup, Denmark) positivity in mirror of epithelial component. Immunophenotyping demonstrated an increase of CD8+ cytotoxic T-lymphocytes (clone* C8/144B, 1 : 200, Dako *Glostrup, Denmark) in predominantly CD3+ T-lymphocytes (clone* LN10, ready to use, Leica* Newcastle, United Kingdom) contingent. PDL 1 (programmed death-ligand 1) (clone* 22C3, ready to use, Dako* Glostrup, Denmark) expression was negative in the epithelial component but scattered lymphocytes in peritumoral infiltrate were positive (Figure 3).

Gene mutation testing has been performed by next generation sequencing (NGS), as we have previously validated, with a panel of 16 genes described in Table 1 [23].

Two mutations were found: K132M mutation of the p53 gene and R88Q mutation of the PIK3CA gene.

In situ hybridization (ISH) with EBER probe for qualitative identification of Epstein-Barr Virus (EBV) using the automated Leica BOND-III system (Leica Biosystems, Nussloch, Germany) was negative [24]. In addition, the detection of High Risk-HPV DNA (HPV 16, 18, 31, 33, 35, 39, 45, 51, 52, 56, 59, 66, and 68) from the paraffin-embedded sample using the BD onclarity HPV assay (BD diagnostics, Sparks, USA) was also negative [25].

According to all these pathological and molecular data, the diagnosis of lymphoepithelioma-like carcinoma was performed. The tumor was limited to the uterus, no lymph node involvement was observed, and therefore it was staged pT1aNo according to the WHO 2014. No complementary treatment was applied and to date with a follow-up of 16 months the patient was disease free.

## 3. Discussion

In the present study, we have demonstrated for the first time some molecular characteristics of lymphoepithelioma-like carcinoma of the endometrium which is an extremely rare uterine tumor [12–16]. In particular, we have demonstrated mutations affecting, respectively, p53 and PIK3CA genes. Classically, according to new molecular subclassification of endometrial carcinoma, mutations of p53 gene were associated with poor prognosis uterine tumors including high grade endometrioid, serous carcinomas, and mixed epithelial and mesenchymal tumors/carcinosarcoma [17–19]. PIK3CA mutations are less specific and encountered in all the four molecular endometrial carcinoma subtypes and to date as in the present case lymphoepithelioma-like carcinoma was not associated with microsatellite instability and loss of the MLH1 protein expression [13].

P53 mutations appeared as common genetic trait in breast medullary carcinoma which showed common features with other lymphoepithelioma-like carcinoma including syncytial sheet of large pleomorphic cells without glandular differentiation and numerous mature lymphocytes and plasma cells in the adjacent stroma [26, 27]. Interestingly, these tumors have been reported to have a better prognosis than common invasive breast carcinoma subsequently firstly to a better response to chemotherapy due to intense mitotic activity and secondly the increase of T-lymphocytes and particularly CD8 cytotoxic T-lymphocytes which play a crucial function in antitumor response [25]. Classically, in high grade endometrioid and serous carcinoma of the endometrium often associated with p53 mutations and a poor clinical outcome, there is a major CD8 downregulation on cytotoxic T-lymphocytes [28]. Therefore, the alterations on cell cycle, apoptosis, and/or senescence consecutive to the loss p53 function by mutations are not counterbalanced by antitumoral immune response. Concerning our patient, even if it is encouraging, the unremarkable follow-up without additional therapy of 16 months is too short to draw any relevant conclusions. Furthermore, studies investigating the immunologic and molecular biomarkers associated with this rare variant of endometrial carcinoma are warranted.

## Figures and Tables

**Table 1:** Cancer hotspot panel used by NGS.

AKT1	DICER1	FOXL2	POLE
BRAF	ERBB2	KRAS	PTEN
CDKN2A	FBXW7	PIK3CA	RB1
CTNNB1	FGFR2	PIK3R1	TP53
